# The morbidity of urethral stricture disease among male Medicare beneficiaries

**DOI:** 10.1186/1471-2490-10-3

**Published:** 2010-02-18

**Authors:** Jennifer T Anger, Richard Santucci, Anna L Grossberg, Christopher S Saigal

**Affiliations:** 1 Department of Urology, David Geffen School of Medicine, University of California, Los Angeles, USA; 2 Department of Urology, Detroit Medical Center; Detroit, Michigan, USA

## Abstract

**Background:**

To date, the morbidity of urethral stricture disease among American men has not been analyzed using national datasets. We sought to analyze the morbidity of urethral stricture disease by measuring the rates of urinary tract infections and urinary incontinence among men with a diagnosis of urethral stricture.

**Methods:**

We analyzed Medicare claims data for 1992, 1995, 1998, and 2001 to estimate the rate of dual diagnoses of urethral stricture with urinary tract infection and with urinary incontinence occurring in the same year among a 5% sample of beneficiaries. Male Medicare beneficiaries receiving co-incident ICD-9 codes indicating diagnoses of urethral stricture and either urinary tract infection or urinary incontinence within the same year were counted.

**Results:**

The percentage of male patients with a diagnosis of urethral stricture who also were diagnosed with a urinary tract infection was 42% in 2001, an increase from 35% in 1992. Eleven percent of male Medicare beneficiaries with urethral stricture disease in 2001 were diagnosed with urinary incontinence in the same year. This represents an increase from 8% in 1992.

**Conclusions:**

Among male Medicare beneficiaries diagnosed with urethral stricture disease in 2001, 42% were also diagnosed with a urinary tract infection, and 11% with incontinence. Although the overall incidence of stricture disease decreased over this time period, these rates of dual diagnoses increased from 1992 to 2001. Our findings shed light into the health burden of stricture disease on American men. In order to decrease the morbidity of stricture disease, early definitive management of strictures is warranted.

## Background

Although the true incidence of urethral stricture disease in men is unknown, Medicare utilization rates for men age 65 and over were 0.9% in 2001, a decrease from 1.4% in 1992 [1]. Utilization data from the Veteran Affairs (VA) in 2003 revealed a prevalence rate of 193 per 100,000 diagnoses (0.2%) [2]. Urethral stricture disease imposes a great burden on both health and quality of life in men. Previous studies of male urethral stricture disease have shown that nearly 90% of men present with complications [2]. The majority of men with a stricture suffer from obstructive and irritative voiding symptoms, and many experience hematuria, recurrent urinary tract infections, and the need for repeated procedures such as dilation or urethrotomy [2-5]. More severe complications, including acute urinary retention, urethral carcinoma, renal failure, Fournier's gangrene, and bladder atonia have been reported in a small minority of men with stricture disease [2].

The therapy for urethral strictures is also known to be associated with complications that further amplify the burden of disease. Investigations on complication rates following urethrotomy have reported bleeding in 4-6% of cases, infection in 8-9%, incontinence in 1%, and impotence in 1% [5-7]. Additionally, strictures tend to recur after urethrotomy or dilation, with success rates varying based on stricture length and location [8,9]. Long-term success of dilation and urethrotomy for longer strictures greater than two centimeters is less than 35% [10]. Men whose strictures recur after a single urethrotomy or dilation are almost invariably likely to fail a second procedure [6,7]. Although definitive urethroplasty has a very high cure rate with acceptably low morbidity, unfortunately this treatment modality is performed infrequently [1].

To better understand the morbidity of urethral stricture disease on American men, we used Medicare claims data in order to measure the true association of male urethral stricture disease with both urinary tract infections and urinary incontinence.

## Methods

And IRB exemption from UCLA was granted for this project, as all data was de-identified. We analyzed Medicare claims data for 1992, 1995, 1998, and 2001 to estimate the rate of dual diagnoses of urethral stricture with urinary tract infection and with urinary incontinence occurring in the same year. The datasets were derived from the Centers for Medicare and Medicaid Services (CMS) administrative records as a 5% random sample, as previously described. Data from three Medicare files, including Medicare Provider Analysis and Review (MEDPAR), carrier, and outpatient, were linked to determine beneficiary use in the inpatient, ambulatory surgery center, hospital outpatient, physician office, and emergency room settings. Estimates for disease prevalence were derived from these data files. For each condition, International Classification of Diseases, 9^th ^Edition (ICD-9) codes for urethral stricture were applied to analytical files from each of the above datasets [2]. Male Medicare beneficiaries receiving co-incident ICD-9 codes indicating diagnoses of urethral stricture and either urinary tract infection or urinary incontinence within the same year were counted. Denominators were derived using the CMS enrollment file, which supplied data on all Medicare beneficiaries enrolled in the years analyzed. Results were stratified by age, race/ethnicity, and geographic region. Because a 5% sample of Medicare records was used, estimates were obtained by multiplying counts by a constant weight of 20. The methodology and analytical methods used to generate these results have been described previously [11].

## Results

The percentage of men with a diagnosis of urethral stricture who were also diagnosed with urinary tract infection in the same year increased from 35% in 1992 to 42% in 2001 (Additional file 1: Table S1). This association was seen across all age groups. Additionally, this association was seen across all races analyzed, with the largest association noted in Hispanic populations (52-56%). Data was also stratified by location, and all regions of the United States were noted to have comparable associations between urethral stricture disease and urinary tract infections.

There was also a significant association of urethral stricture disease with urinary incontinence. Eleven percent of male Medicare beneficiaries with urethral stricture disease in 2001 were diagnosed with urinary incontinence in the same year (Additional file 2: Table S2). This represents an increase from 8% in 1992. This association spanned all age ranges of male Medicare beneficiaries, with increased associations seen with increasing age. Among all races analyzed, Hispanics with stricture disease had the highest rate of associated urinary incontinence (18%). There was no regional variation appreciated.

## Discussion

Although urethral stricture disease is known to be associated with other urologic conditions, the association of urinary tract infection and urinary incontinence with urethral strictures has been largely undefined in large series. The data presented in this investigation provide evidence of a strong association between male urethral stricture disease and urinary tract infections, occurring as a co-diagnosis in 41% of male Medicare beneficiaries. The association between male urethral stricture disease and urinary incontinence is also significant, as demonstrated by a co-diagnosis in 11% of Medicare beneficiaries with a diagnosis of urethral stricture disease.

An association of urinary tract infections with urethral strictures has been previously documented. Romero et al analyzed the presentation of 175 patients with urethral stricture disease, of which sixty-three (36%) presented with urinary tract infection [12]. Urethral strictures incite a condition of urinary stasis, of which urinary tract infection is a known outcome secondary to an increased post-void residual volume [12]. Frequent instrumentation employed in the diagnosis and management of urethral stricture disease is another potential cause for infection, with introduction of organisms in a retrograde fashion through the urethra, which may then colonize the lower urinary tract.

The association between urethral strictures and urinary incontinence has been previously documented but not well quantified. Following procedures for proximal bulbar urethral stricture disease adjacent to the external sphincter, incontinence is a known potential adverse outcome in men who have undergone previous procedures involving the bladder neck, such as radical prostatectomy, transurethral resection of the prostate, or bladder neck incision. The reason for increasing rates of both urinary tract infections and urinary incontinence over the 1992-2001 time period may be due to new treatments for BPH and prostate cancer that have a more severe effect on the urethra.

Overall rates of stricture prevalence decreased over the 1992-2001 time period, possibly due to lower rates of infectious urethritis, as well as better cure rates of long strictures with the use of urethroplasty with buccal graft augmentation [2,13,14]. However, the rate of dual diagnoses of stricture with UTI and incontinence increased over this time period. This may possibly be due to a shift in stricture characteristics toward a more aggressive stricture caused by one of many available treatments for prostate cancer or BPH. Urethral stricture disease occurs in up to 8.4% of men after treatment for prostate cancer [15]. This includes men who undergo radical prostatectomy, as well as men who undergo non-surgical therapy such as conformal beam radiation, seed implantation, and cryotherapy. The tissue effects of such non-surgical treatments can often cause strictures involving the proximal bulbar and membranous urethra. At the same time, the bladder neck becomes rigid and immobile. Hence, procedures performed to open a proximal bulbar urethral stricture may result in incontinence when the bladder neck is no longer competent.

Medicare claims data are particularly useful in that they allow for the estimation of the rates of diagnoses across a large, heterogeneous, nationwide sample of the elderly population across various clinical settings. However, the use of claims data has several limitations. Medicare claims provide limited clinical information, and we were therefore unable to demonstrate a causal relationship between urethral stricture disease and the other urologic conditions investigated. Additionally, we do not have data indicating whether patients had previously received radiation or other therapy for prostate cancer, which further complicates our ability ascertain whether incontinence occurred strictly as a result of stricture disease. Furthermore, examining claims diagnoses may be inherently inaccurate in some cases. For example, bladder neck contractures (ICD-9 code 596.0) may be mislabeled as urethral strictures in coding, which may affect our findings. Although not a true urethral stricture, a bladder neck contracture occurring after radical prostatectomy is highly associated with incontinence [16]. Opening a bladder neck contracture with a bladder neck incision often unmasks incompetence of the external urethral sphincter. Hence, coding a bladder neck contracture as a urethral stricture may have caused us to overestimate the true association of stricture disease with incontinence. Finally, Medicare datasets reflect diagnoses among men age 65 and over. Our findings therefore may not be generalizable to younger men with strictures.

## Conclusions

Among male Medicare beneficiaries diagnosed with urethral stricture disease in 2001, 42% were also diagnosed with a urinary tract infection, and 11% with incontinence. Although the overall incidence of stricture disease decreased over this time period, these rates of dual diagnoses increased from 1992 to 2001, possibly relating to newer and more aggressive treatments for prostate cancer and BPH. Our findings shed light into the health burden of stricture disease on American men. Early urethroplasty has the potential to cure anterior urethral strictures early, significantly decreasing the morbidity of stricture disease described in this paper.

## Competing interests

The authors declare that they have no competing interests.

## Authors' contributions

JA and AG participated in the design of the study, performed the statistical analysis, and wrote the manuscript. RS and CS conceived of the study, and participated in its design and coordination. They also reviewed and revised the manuscript. All authors read and approved the final manuscript.

## Pre-publication history

The pre-publication history for this paper can be accessed here:

http://www.biomedcentral.com/1471-2490/10/3/prepub

## Supplementary Material

Additional file 1 **Table S1**. Male Medicare beneficiaries with a diagnosis of urethral stricture and urinary tract infection (UTI) in the same year, count, percent. Click here for file

Additional file 2 **Table S2**. Male Medicare beneficiaries with a diagnosis of urethral stricture and urinary incontinence in the same year, count, percent. Click here for file
