# Supplementary material for: The morbidity of urethral stricture disease among male Medicare beneficiaries
Source: BMC Urol. 2010 Feb 18;10:3. doi: 10.1186/1471-2490-10-3 (PMC2837655; doi:10.1186/1471-2490-10-3)
Supplement: Additional file 1 — Table S1. Male Medicare beneficiaries with a diagnosis of urethral stricture and urinary tract infection (UTI) in the same year, count, percent. [file 1471-2490-10-3-S1.PDF]

Table 1. Male Medicare beneficiaries with a diagnosis of urethral stricture and urinary tract infection (UTI) in the same year, count[a], percent[b]

	1992		1995		1998		200[illegible]
	Count	Percent	Count	Percent	Count	Percent	Count
Total[c]	58,040	35	54,020	37	49,560	42	46,400
Age							
65–69	10,440	31	9,560	34	7,840	38	7,320
70–74	13,400	32	12,680	35	11,000	39	10,780
75–79	14,660	36	13,180	36	12,640	42	11,480
80–84	10,660	37	10,640	38	9,740	44	8,620
85–89	6,280	43	5,420	42	6,120	47	5,780
90–94	2,060	45	2,080	44	1,780	51	2,100
95–97	320	48	360	58	320	43	220
98+	220	58	100	56	120	75	100
Race/ethnicity							
Asian	…	…	200	26	280	37	600
Black	7,680	44	5,920	40	5,460	46	4,760
Hispanic	…	…	900	52	1,640	56	1,520
N. American Native	…	…	80	67	20	20	20
White	47,140	34	45,740	36	41,400	41	38,520
Region							
Midwest	14,400	34	13,900	36	12,380	39	10,780
Northeast	10,520	33	9,300	34	9,860	44	8,380
South	24,960	38	22,120	37	19,120	42	18,260
West	7,280	33	7,480	38	6,720	41	7,520

…data not available.

[a]Unweighted counts multiplied by 20 to arrive at values in the table.

[b]Percent of males in each cell who had UTI diagnosis.

[c]Males of other races, unknown race and ethnicity, and other region are included in the tables.

SOURCE: Centers for Medicare and Medicaid Services, 1992, 1995, 1998, 2001.

)1
Percent
42
40
42
42
41
45
49
44
71
46
44
52
20
42
37
43
43
44
