# Supplementary material for: The morbidity of urethral stricture disease among male Medicare beneficiaries
Source: BMC Urol. 2010 Feb 18;10:3. doi: 10.1186/1471-2490-10-3 (PMC2837655; doi:10.1186/1471-2490-10-3)
Supplement: Additional file 2 — Table S2. Male Medicare beneficiaries with a diagnosis of urethral stricture and urinary incontinence in the same year, count, percent. [file 1471-2490-10-3-S2.PDF]

Table 2. Male Medicare beneficiaries with a diagnosis of urethral stricture and urinary incontinence in the same year, count[a], percent[b]

	1992		1995		1998		200
	Count	Percent	Count	Percent	Count	Percent	Count
Total[c]	13,220	8	11,940	8	11,200	9	11,760
Age							
65–69	1,680	5	1,880	7	1,660	8	1,440
70–74	3,160	8	2,860	8	2,320	8	2,600
75–79	3,020	7	2,700	7	3,000	10	2,620
80–84	2,960	10	2,660	10	2,160	10	2,700
85–89	1,600	11	1,180	9	1,640	13	1,580
90–94	660	14	540	11	340	10	740
95–97	100	15	100	16	80	11	60
98+	40	11	20	11	0	0	20
Race/ethnicity							
Asian	…	…	40	5	100	13	240
Black	1,580	9	1,060	7	1,120	10	1,440
Hispanic	…	…	60	3	280	10	260
N. American Native	…	…	0	0	0	0	20
White	10,900	8	10,560	8	9,560	9	9,560
Region							
Midwest	3,860	9	3,120	8	2,940	9	2,680
Northeast	1,880	6	2,180	8	2,240	10	2,340
South	5,360	8	4,980	8	4,400	10	4,720
West	2,060	9	1,520	8	1,500	9	1,840

…data not available.

[a]Unweighted counts multiplied by 20 to arrive at values in the table.

[b]Percent of males in each cell who have UI diagnosis.

[c]Males of other races, unknown race and ethnicity, and other region are included in the tables.

SOURCE: Centers for Medicare and Medicaid Services, 1992, 1995, 1998, 2001.

1
Percent
11
8
10
10
13
12
17
12
14
18
13
9
20
10
9
12
11
11
